# A molecular phylogeny of the spiny lobster *Panulirus homarus* highlights a separately evolving lineage from the Southwest Indian Ocean

**DOI:** 10.7717/peerj.3356

**Published:** 2017-05-25

**Authors:** Sohana P. Singh, Johan C. Groeneveld, Abdulaziz Al-Marzouqi, Sandi Willows-Munro

**Affiliations:** 1 Oceanographic Research Institute, Durban, KwaZulu-Natal, South Africa; 2 School of Life Sciences, University of KwaZulu-Natal, Pietermaritzburg, KwaZulu-Natal, South Africa; 3 Marine Science and Fisheries Centre, Ministry of Agriculture and Fisheries, Muscat, Oman

**Keywords:** Decapoda, Spiny lobster, Phylogeny, Species concept, Coalescence, Divergence dating

## Abstract

Accurate species description in the marine environment is critical for estimating biodiversity and identifying genetically distinct stocks. Analysis of molecular data can potentially improve species delimitations because they are easily generated and independent, and yield consistent results with high statistical power. We used classical phylogenetic (maximum likelihood and Bayesian inference) and coalescent-based methods (divergence dating with fossil calibrations and coalescent-based species delimitation) to resolve the phylogeny of the spiny lobster *Panulirus homarus* subspecies complex in the Indo-West Pacific. Analyses of mitochondrial data and combined nuclear and mitochondrial data recovered *Panulirus homarus homarus* and *Panulirus homarus rubellus* as separately evolving lineages, while the nuclear data trees were unresolved. Divergence dating analysis also identified *Panulirus homarus homarus* and *Panulirus homarus rubellus* as two distinct clades which diverged from a common ancestor during the Oligocene, approximately 26 million years ago. Species delimitation using coalescent-based methods corroborated these findings. A long pelagic larval life stage and the influence of ocean currents on post-larval settlement patterns suggest that a parapatric mode of speciation drives evolution in this subspecies complex. In combination, the results indicate that *Panulirus homarus rubellus* from the Southwest Indian Ocean is a separately evolving lineage and possibly a separate species.

## Introduction

What constitutes a species or subspecies? In light of conflicting hypotheses regarding species concepts, this is a difficult question to answer. Whereas all species concepts accept that a species is a separately evolving metapopulation lineage (Agapow et al., 2004; de Queiroz, 2007), secondary criteria differ. For instance, the biological concept states that there must be reproductive isolation from other lineages (Mayr, 1942), while the phylogenetic concept proposes that a lineage must be monophyletic to qualify as a species (Cracraft, 1983). Furthermore, all the secondary characteristics that define lineage diversification don’t necessarily occur at the same time or linearly (de Queiroz, 2007), and as a result, organisms might be classified as a subspecies when they are in fact a recently diverged species (Parkin & Knox, 2010). Subspecies are valuable to the studies of biodiversity and evolution, as they reflect the earliest stages of speciation (Johnsen et al., 2006).

The advent of molecular data has made it possible to test traditional subspecies delineations (Ball & Avise, 1992; Barrowclough, 1980; Burbrink, Lawson & Slowinski, 2000; Morin et al., 2010; Phillimore & Owens, 2006). Statistical power and rigor of methods and algorithms used for the molecular delimitation of species are constantly improving and yielding consistent results (de Queiroz & Gatesy, 2007; Rannala & Yang, 2013). In addition to classical multi-locus phylogenetic methods, coalescent-based species delimitations using molecular data have been applied successfully in many studies (Burbrink et al., 2011; Leache & Fujita, 2010; Setiadi et al., 2011; Zhang, Zhang & Yang, 2011), and are useful for identifying species that have recently diverged or are in the process of divergence (Knowles & Carstens, 2007). Using coalescent theory (Hudson, 1991; Kingman, 1982) and applying the general lineage concept (de Queiroz, 2007), probabilities for allele sorting under alternative hypotheses can be calculated. The shared ancestral polymorphisms detected using the genetic data and coalescent methods can enable species detection, or a lineage split, at the early stage of divergence, before monophyly (Knowles & Carstens, 2007).

Marine organisms such as spiny lobsters (Palinuridae) are good models for the study of speciation and the validity of subspecies because of their high dispersal capabilities (Palumbi, 1994). Spiny lobsters have high fecundity and long-lived phyllosoma larvae that drift in the water column for several months, with the potential to disperse over long distances (summarized by George, 2005). This in turn promotes large populations, large geographic ranges and enables high levels of gene flow.

The earliest lineages of lobsters from all infraorders originated approximately 360 million years ago (MYA), during the late Devonian period in the Paleozoic era (Bracken-Grissom et al., 2014; Schram & Dixon, 2004). The Achelata infraorder diverged into the spiny (Palinuridae) and slipper lobster (Scyllaridae) families around 250 MYA (Bracken-Grissom et al., 2014; George, 2006; Tsang et al., 2009). These authors propose that, approximately 230 MYA, the Palinuridae diverged into stridulating (sound-producing) Stridentes (*Linuparus*, *Justitia*, *Nupalirus*, *Palinustus*, *Puerulus*, *Palibythus, Palinurus* and *Panulirus*) and non-stridulating Silentes groups (*Projasus*, *Jasus*, *Sagmariasus* and *Palinurellus*). Within the Stridentes, the shallow warm water *Panulirus* genus is probably the most recently evolved (George & Main, 1967; George, 2006, 1997; Pollock, 1992). This is supported by a molecular phylogenetic study on the genus (Ptacek et al., 2001) and another study using fossil calibrated data in conjunction with molecular DNA markers, which showed that *Panulirus* emerged around 160 MYA (Bracken-Grissom et al., 2014). A conflicting hypothesis by Tsang et al. (2009), based on protein-coding molecular data, suggests that *Panulirus* is basal in the Stridentes group.

The scalloped spiny lobster *Panulirus homarus* comprises three economically important subspecies in the Indo-West Pacific region, extending northwards from Southeast Africa and Madagascar, along the coast of the Western Indian Ocean to the Arabian Sea and India in the north, and along the western rim of the Pacific, to Indonesia, Japan and Australia (Holthuis, 1991). The three subspecies are phenotypically distinguishable and their geographical ranges differ. The nominotypical *Panulirus homarus homarus* has small squamae on the abdominal segments (microsculpta), is dark green in color, and occurs throughout the Indo-West Pacific (Berry, 1971; Holthuis, 1991; Lavery et al., 2014). *Panulirus homarus megasculptus* has large squamae (megasculpta), is olive green with yellow lateral markings, and appears to be restricted to the Northern Arabian Sea. *Panulirus homarus rubellus* is the red megasculpta form, which occurs in the Southwest Indian Ocean, along the coasts of eastern South Africa, Mozambique and Southern Madagascar.

Three molecular studies have been done on *Panulirus homarus* and its subspecies. Nuclear copies of mitochondrial DNA (numts or pseudogenes) COI data showed that there is significant genetic partitioning between *Panulirus homarus rubellus* from Southeast Madagascar and those from the African shelf, which suggests the Mozambique Channel as a barrier to larval dispersal (Reddy et al., 2014). *Panulirus homarus* samples from Tanzania and the Arabian Sea belonged to different stocks, likely because of the effects of local currents on larval dispersal (Farhadi et al., 2013). Using the genetic markers COI, control region (CR), 18S rDNA and the ITS-1 intron, Lavery et al. (2014) found little genetic differentiation between the *Panulirus homarus homarus* and *Panulirus homarus megasculptus* sub-species, which indicates that *Panulirus homarus megasculptus* should not be considered a separate subspecies. *Panulirus homarus rubellus* was the most divergent subspecies, but a single observation of hybridization between *Panulirus homarus homarus* and *Panulirus homarus rubellus* suggested that interbreeding may occur.

We used multilocus genetic data from mitochondrial (COI and hypervariable control region) and nuclear (ITS-1 intron and β-tubulin) markers, and employed both classical phylogenetic (Bayesian inference (BI) and maximum likelihood (ML)) and coalescent-based methods to resolve the phylogeny of the *Panulirus homarus* subspecies complex. Fossil data was used to infer divergence times between the *Panulirus homarus* subspecies. Our study extends the work done by Lavery et al. (2014) on *Panulirus homarus* by analyzing a concatenated multi-marker dataset, and using additional coalescent-based methods and fossil data to better understand the evolution of the subspecies complex.

## Materials and Methods

### Sample collection

**Figure 1:**
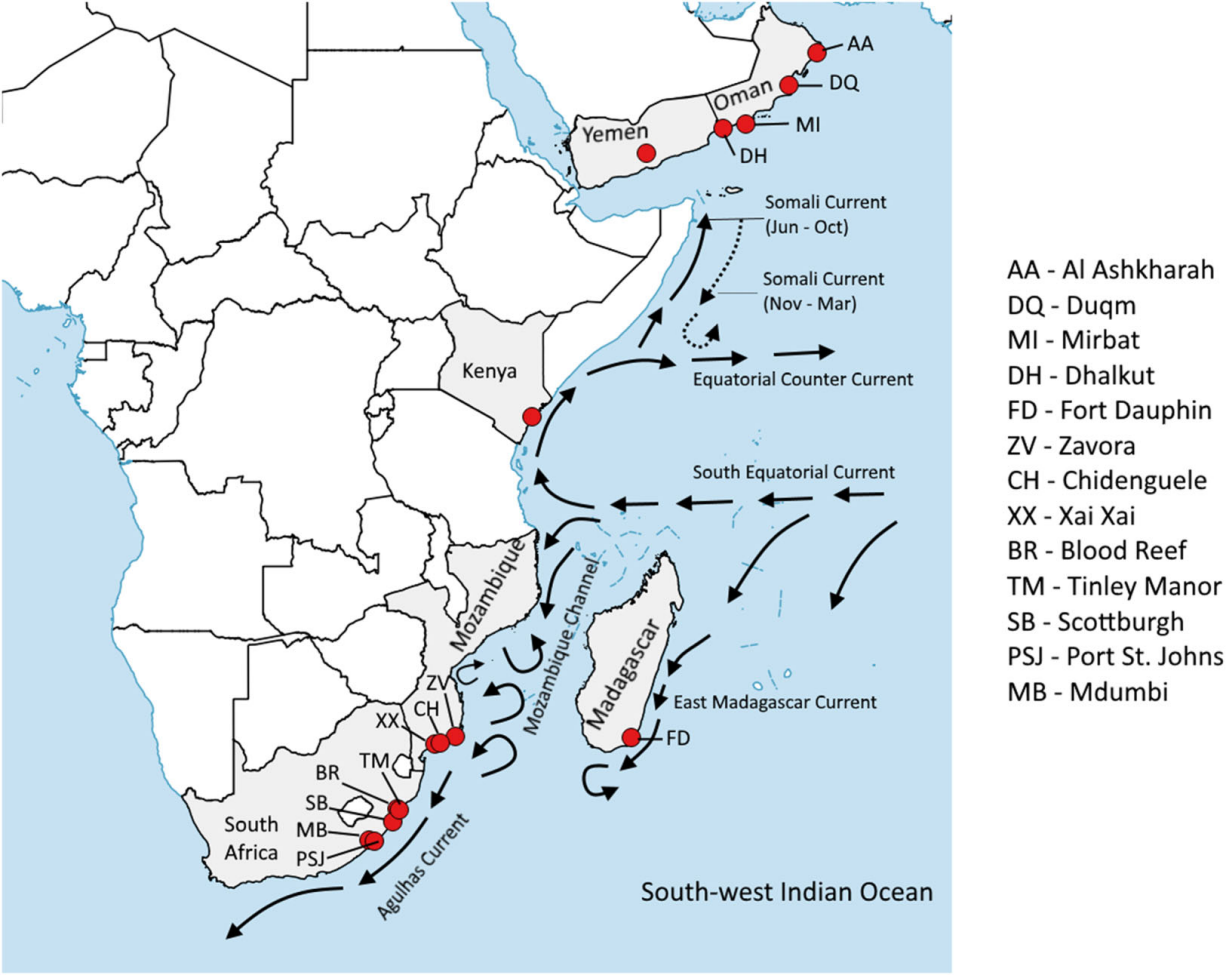
Sampling sites of the *Panulirus homarus* subspecies. The main ocean currents and eddy systems are depicted (adapted from Lutjeharms, 2006).

*Panulirus homarus* specimens were collected from five sites along the east coast of South Africa (Tinley Manor, Blood Reef, Scottburgh, Mdumbi and Port St. Johns), three sites in Mozambique (Chidenguele, Xai Xai and Zavora) and one site in Madagascar (Fort Dauphin). Additional samples were sourced from four sites in Oman (Al Ashkharah, Dhalkoot, Duqm and Mirbat), and one site each in Yemen and Kenya (Fig. 1). All specimens were identified to subspecies level based on phenotypic and geographic information.

### DNA extraction, PCR amplification and sequencing

DNA was extracted from pereiopod tissue using the Zymo ZR Tissue and Insect DNA kit (Inqaba Biotec, Hatfield, Pretoria, South Africa), as per the manufacturers protocol which was modified slightly to replace the bead bashing process with the addition of 15 μl of Proteinase K and a 3-h incubation at 56 °C during the lysis step.

Molecular markers used in the study included mitochondrial COI (LCO-Ph 5′-CGGAGCATGAGCTGGGATAGT-3′ and HCO-Ph 5′-ACTTCTGGGTTGTCGAGGACTC-3′; Lavery et al., 2014) and CR (CR1 5′-GCA AAG AAT ATA GCA AGA ATC AA-3′ and CR2 5′-GCA AAC CTT TTT ATC AGG CAT C-3′; Diniz et al., 2005). Nuclear markers included ITS-1 (ITSF 5′-CACACCGCCCGTCGCTACTA-3′ and ITSR 5′-ATTTAGCTGCGGTCTTCATC-3′; Chu, Li & Ho, 2001) and β-tubulin (BTF2 5′-ATGTTYGAYGCHAAGAAYATGATGGC-3′ and BTR2 5′-TCCATGCCYTCNCCVGTGTACCAGTG-3′; Jennings & Etter, 2011). Amplification reactions were 25 μl in total and contained 2 μl of 10× PCR reaction buffer (Super-Therm®; Industricord, Gillitts, South Africa), 2 μM MgCl_2_, 0.2 μM of dNTP mix, 0.2 μM of each 10 μM primer and 0.2 μl of 1 U Taq polymerase (Super-Therm®; Industricord, Gillitts, South Africa).

The thermal cycling program for all markers consisted of initial denaturation at 95 °C for 10 min, followed by 35 cycles of 95 °C for 30 s, annealing temperatures of 50 °C (COI), 57.4 °C (ITS-1 and CR) and 54 °C (β-tubulin) for 30 s, and 72 °C for 45 s. The final extension step was carried out at 72 °C for 10 min. All PCR reactions were run with a positive and negative control.

PCR clean-up and sequencing reactions were performed at the central analytical facilities at Stellenbosch University. Chromatograms were assembled and checked manually using BioEdit v. 7.2.5 (Hall, 1999) and FinchTV v. 1.4.0 (http://www.geospiza.com/). Multiple sequence alignment was done using the online version of MAFFT (Katoh et al., 2002) and then refined manually. The Gblocks server (http://molevol.cmima.csic.es/castresana/Gblocks_server.html, v. 0.91b) was used to assess the confidence of the final alignments. The strict parameter of not allowing many contiguous non-conserved positions was chosen, but gap positions were allowed within final blocks. Additionally, the GUIDANCE2 server (http://guidance.tau.ac.il/) was used to calculate confidence scores for each alignment. The COI sequences were checked for stop codons by using the NCBI ORF Finder (Wheeler et al., 2003) and translation to protein to ensure that a pseudogene was not being amplified. Nuclear data were phased using Seqphase (Flot, 2009) and PHASE v. 2.1 (Stephens, Smith & Donnelly, 2001) to investigate the occurrence of hybridization between the sub-species.

In addition to *Panulirus homarus* individuals, DNA was also extracted from eight other lobster species; *Jasus paulensis*, *Jasus lalandii*, *Palinurus gilchristi*, *Palinurus delagoae*, *Panulirus longipes*, *Panulirus versicolor*, *Scyllarides elisabethae* and *Scyllarides squammosus*. The four markers were also amplified in these individuals for use as outgroup taxa and fossil calibration points for divergence dating analysis. All sequences used in this study are listed with their accession numbers in Table S1.

### Phylogenetic analyses

To infer the phylogeny of *Panulirus homarus*, a ML approach implemented in Garli v. 2.0 (Zwickl, 2006), and BI approach implemented in MrBayes v. 3.2.6 (Huelsenbeck & Ronquist, 2005; Ronquist & Huelsenbeck, 2003), were used. The best models of nucleotide substitution for each gene were selected using jModeltest v. 2.0 (Darriba et al., 2012) and the corrected Akaike information (AICc) criterion (Table 1). Each gene was analyzed separately and then combined by genomic location; mitochondrial (COI + CR) and nuclear (β-tubulin + ITS-1), and then concatenated into a single dataset (COI + CR + β-tubulin + ITS-1) in SequenceMatrix v. 1.8 (Vaidya, Lohman & Meier, 2011). PartitionFinder v. 1.1.1 (Lanfear et al., 2012) was used to find the best partitioning strategy and model for each codon position in COI and for each partition in the concatenated datasets (mitochondrial, nuclear and the four-genes concatenated).

**Table 1:** Sequence alignment characteristics and best models for nucleotide sequence evolution for datasets used in the analyses.

Marker	Sites	N	Variable	Parsimony informative	Model
COI	565	79	165	95	cp01: TIMef + G, cp02: F81 + I, cp03: GTR + G
CR	541	55	184	142	TVM + I + G
β-tubulin	264	54	98	75	TVMef + I + G
ITS-1	437	61	231	145	TIMef + I + G
Mitochondrial	1,106	55	314	214	*
Nuclear	701	47	280	198	*
Concatenated	1,807	54	650	425	*

**Note:**

* Individual models for each gene were used in the combined datasets.

The Garli search was performed using two independent runs with two search replicates each. Nodal support was assessed by a 1,000 bootstrap replicates. The number of generations run in the BI analysis was 20,000,000 for COI, CR and β-tubulin, and 50,000,000 for ITS-1, the combined mitochondrial, combined nuclear and the four-genes concatenated. Two independent runs with four parallel Markov Chain Monte Carlo (MCMC) chains were performed for each of the datasets. Trees were sampled every 1,000 generation. The number of trees to be discarded as burn-in and effective sample size (ESS) values to check for MCMC convergence was assessed using Tracer v. 1.6 (Rambaut & Drummond, 2007). ESS values that were greater than 200 indicated that there was chain convergence, and that the analysis was run long enough to obtain valid estimates of the parameters. Bootstrap values and posterior probabilities were mapped on to the most likely tree for each gene, the combined mitochondrial, combined nuclear and all four genes concatenated. The analyses for each of the genes was also performed excluding the outgroups and using the midpoint rooting method to see if the choice of outgroups had any effects on bootstrap and Bayesian posterior probability (BPP) support of ingroups. Genetic distances (*p*-distance) were calculated in MEGA v. 6.0 (Tamura et al., 2013).

### Molecular divergence dating

Divergence dates were estimated using a reduced four gene concatenated dataset which contained 14 *Panulirus homarus homarus* individuals (Kenya: seven and Mozambique: six), 30 *Panulirus homarus rubellus* individuals (South Africa: 16, Mozambique: 11 and Madagascar: three) and nine *Panulirus homarus megasculptus* individuals (Oman: seven and Yemen: two). All taxa had sequence data for at least three markers. Analysis was performed using an uncorrelated Bayesian relaxed molecular clock approach in BEAST 2.4.0 (Bouckaert et al., 2014). In order to introduce fossil calibration points, slipper lobsters from the family *Scyllarides* (*Scyllarides elisabethae* and *Scyllarides squammosus*), spiny lobsters from the *Jasus* (*Jasus paulensis* and *Jasus lalandii*) and *Palinurus* genera (*Palinurus gilchristi*) and two other *Panulirus* species (*Panulirus longipes* and *Panulirus versicolor*) were added to the dataset. The fossil calibration points used, along with the offset and standard deviations, are given in Table 2. The substitution models chosen were the same as those used in the ML and BI analyses. The Yule speciation model was chosen as the tree prior, because it is appropriate for describing the relationships between individuals from different species (Aldous, 2001). Divergence dates were estimated using an uncorrelated relaxed lognormal Bayesian molecular clock. The analysis consisted of two independent MCMC analyses. The chains ran for 70,000,000 generations and trees were sampled every 10,000 generations. Tracer was used to check that the ESS values were greater than 200, confirming good mixing and convergence of the chains. The two runs were combined using LogCombiner v. 2.4.0 and the trees were summarized using TreeAnnotator v. 2.4.0 (included with the BEAST package). A maximum clade credibility consensus tree with mean node heights and posterior probabilities greater than 0.5 was obtained using TreeAnnotator v. 2.4.0. A geological timescale tree was plotted using the packages strap (Bell & Lloyd, 2014), coda (Plummer et al., 2006), phyloch (Heibl, 2008) and phytools (Revell, 2012) in the R statistical package v. 3.1.2 (R Development Core Team, 2008).

**Table 2:** Fossil calibration points used for the divergence dating analysis.

Fossil	Node	Timescale (MYA)	Offset	Standard deviation	Reference
*Yunnanopalinura schrami*	Achelata	241–247	241	0.9	Feldman et al. (2012)
*Archaeopalinurus*	Palinuridae	210–221	210	0.7	Pinna (1974)
*Panulirus destombesi*	Panulirus	99–112	99	0.8	Garassino & Teruzzi (1993)
*Jasus flemingi*	Jasus	5.3–23.8	5.3	0.98	Glaessner (1960)
*Scyllarides bolcensis*	Scyllarides	33.7–54.8	33.7	1	De Angeli & Garassino (2008)

### Molecular species delimitation

The reversible-jump Bayesian Markov Chain Monte Carlo (rjMCMC) algorithm implemented in BP&P v. 3.2 (Bayesian Phylogenetics and Phylogeography; Yang & Rannala, 2010; Yang, 2015; Rannala & Yang, 2013) was used to analyse phylogenetic data from the four loci to generate speciation probabilities based on the multispecies coalescent model. This model takes into account the coalescent processes in the ancestral and the modern species and the resulting gene-species tree conflicts (Degnan & Rosenberg, 2009). The reduced four gene concatenated dataset was used, and the maximum clade probability tree from BEAST was used as the initial guide tree.

The prior settings were as follows: (1) *θ* = G (2, 10) and *τ*_0_ = G (2, 10) for large ancestral population size and deep divergence; (2) *θ* = G (2, 2,000) and *τ*_0_ = G (2, 2,000) for small ancestral populations and shallow divergence; (3) *θ* = G (2, 10) and *τ*_0_ = G (2, 2,000) accounting for large ancestral populations and shallow divergence and; (4) *θ* = G (2, 2,000) and *τ*_0_ = G (2, 10) for small ancestral population size and deep divergence. Algorithm 1 (species delimitation using a fixed guide tree) was used. In this model the rjMCMC algorithm jumps between various species delimitation models compatible with the guide tree supplied (Rannala & Yang, 2013; Yang & Rannala, 2010). The analysis was run twice to confirm stability between runs, each run consisted of 100,000 steps, sampling every 1,000 generations and 8,000 trees were discarded as burnin. Tracer was used to confirm convergence of the chains.

## Results

Amplification was successful for all four markers used in this study, and for the taxa listed in Table S1. The alignment for each locus received a confidence score of >0.98 using the GUIDANCE2 server. The final sequence alignments generated for each marker included (Table 1): COI, 565 bp (193 variable sites); CR, 544 bp (185 variable sites); β-tubulin, 264 bp (154 variable sites) and ITS-1, 444 bp (253 variable sites). The nuclear marker β-tubulin exhibited the most variability (58% variable characters), followed by the nuclear marker ITS-1 (57% variable sites) and mitochondrial CR (34% variable sites) and COI (29% variable sites).

The best-fit model for nucleotide sequence evolution for each of the datasets are shown in Table 1. For the combined datasets, PartitionFinder found the same models as jModeltest for each of the partitions. The ML and BI analyses of the independent datasets, datasets combined by genomic location and the four-gene concatenated dataset, recovered similar topologies with BPP support often being higher than ML bootstrap support. Independent analysis of the four markers revealed no significant conflict (ML bootstrap >50%, BPP >50%, Figs. S1–S4). There was also no conflict between the combined mitochondrial and combined nuclear trees (Figs. S5 and S6).

**Figure 2:**
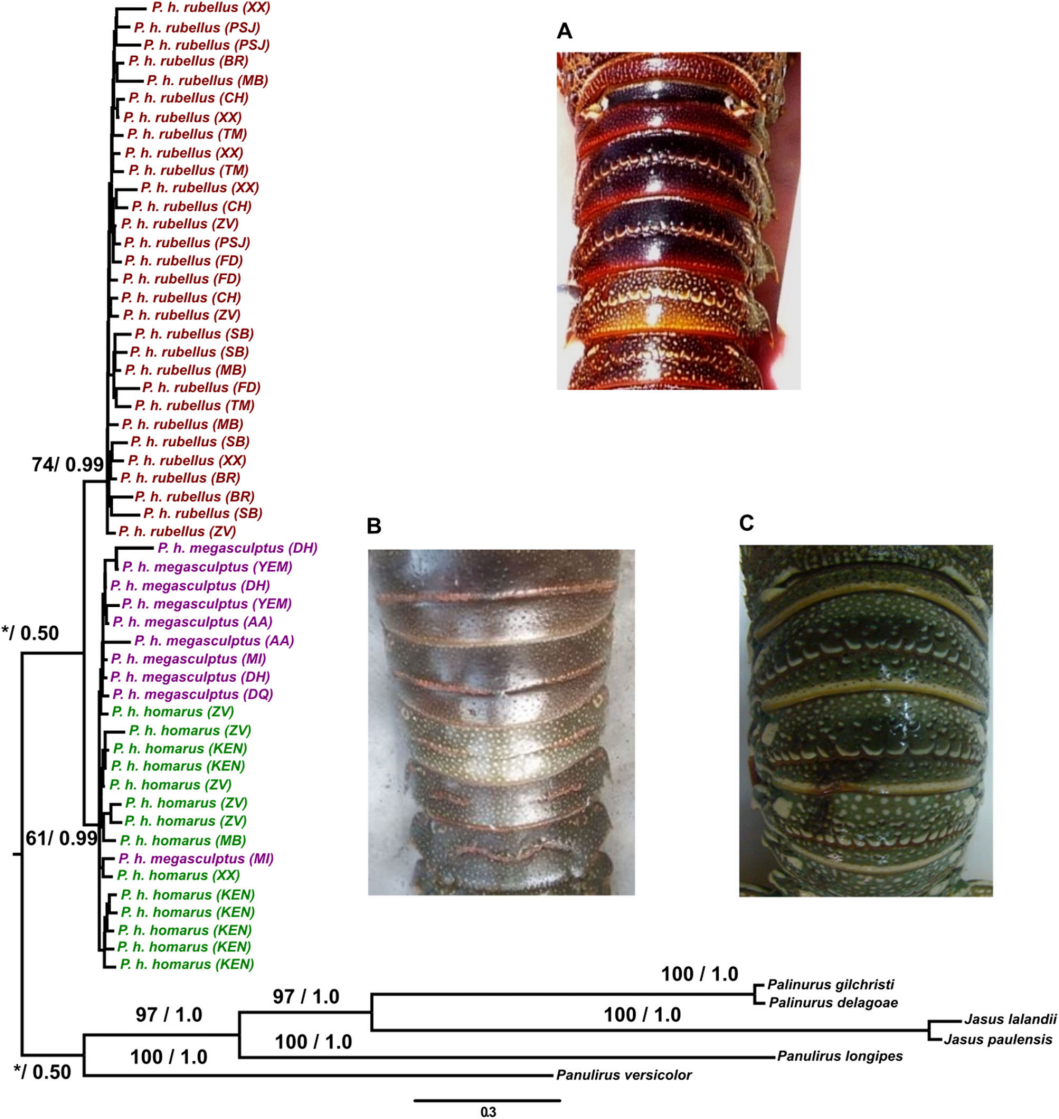
Four gene concatenated tree Maximum likelihood tree inferred from the supermatrix (COI + CR + β-tubulin + ITS-1) data. Maximum likelihood bootstrap support values and Bayesian posterior probabilities are indicated on the nodes. Each color represents the different subspecies. Photograph representatives of each subspecies are shown: (A) *Panulirus homarus rubellus*, (B) *Panulirus homarus homarus* and (C) *Panulirus homarus megasculptus*.

The mtDNA markers, analyzed separately, with outgroup rooting, resulted in two distinct groupings supported by low ML bootstrap support and high BPP support. One group consisted of *Panulirus homarus rubellus* individuals (COI, ML bootstrap: <50%, BPP: 0.95; CR, ML bootstrap: <50%, BPP: 0.73) and the other of *Panulirus homarus homarus* and *Panulirus homarus megasculptus* individuals (COI, ML bootstrap: <50%, BPP: 0.94; CR, ML bootstrap: <50%, BPP: 0.85). The analysis of the combined mtDNA datasets also recovered the two groupings, *Panulirus homarus rubellus* (ML bootstrap: <50%, BPP: 0.83) and *Panulirus homarus homarus* and *Panulirus homarus megasculptus* (ML bootstrap: <50%, BPP: 0.94). The midpoint-rooted individual gene trees for COI and CR, and the combined mtDNA dataset resulted in better ML bootstrap support for *Panulirus homarus rubellus* as a separately evolving group (COI, ML bootstrap: 60%, BPP: 0.95; CR, ML bootstrap: 85%, BPP: 1.0; Combined, ML bootstrap: 74%, BPP: 1.0), and strong support for the *Panulirus homarus homarus* and *Panulirus homarus megasculptus* grouping in the COI data (ML bootstrap: 98%, BPP: 0.9) but weak support in CR data (ML bootstrap: <50%, BPP: <0.5) and the combined mtDNA dataset (ML bootstrap: <50%, BPP: <0.5). The nuclear DNA gene trees (separate and combined, analyzed with and excluding outgroups) were largely unresolved, possibly due to incomplete lineage sorting, slow mutation rates and insufficient informative variation (McCracken & Sorenson, 2005). The four-gene concatenated analysis of all the data, however, resulted in two monophyletic lineages, one containing *Panulirus homarus rubellus* individuals (ML bootstrap: 74%, BPP: 0.99, Fig. 2) and the other *Panulirus homarus homarus* and *Panulirus homarus megasculptus* individuals (ML bootstrap: 61%, BPP: 0.99, Fig. 2).

Uncorrected mean pairwise genetic distances between subspecies and between outgroups were calculated for each marker. Genetic distances calculated for COI and CR were greater between subspecies and between outgroups than distances within subspecies and within outgroups. Interestingly in the β-tubulin gene, the pairwise distance between *Panulirus homarus homarus* and *Panulirus homarus megasculptus* (8.6%, Table S4) was slightly larger than the distance between *Panulirus homarus rubellus* and *Panulirus homarus megasculptus* (8.3%, Table S4). This contrasts with the pairwise distances between the three subspecies for the mitochondrial genes which was 4.8% for COI and 25.8% for CR; and 1.6% between *Panulirus homarus homarus* and *Panulirus homarus megasculptus* for COI and 3.5% for CR (Tables S2 and S3). The genetic distances along with their standard error estimates are included in Tables S2–S5.

**Figure 3:**
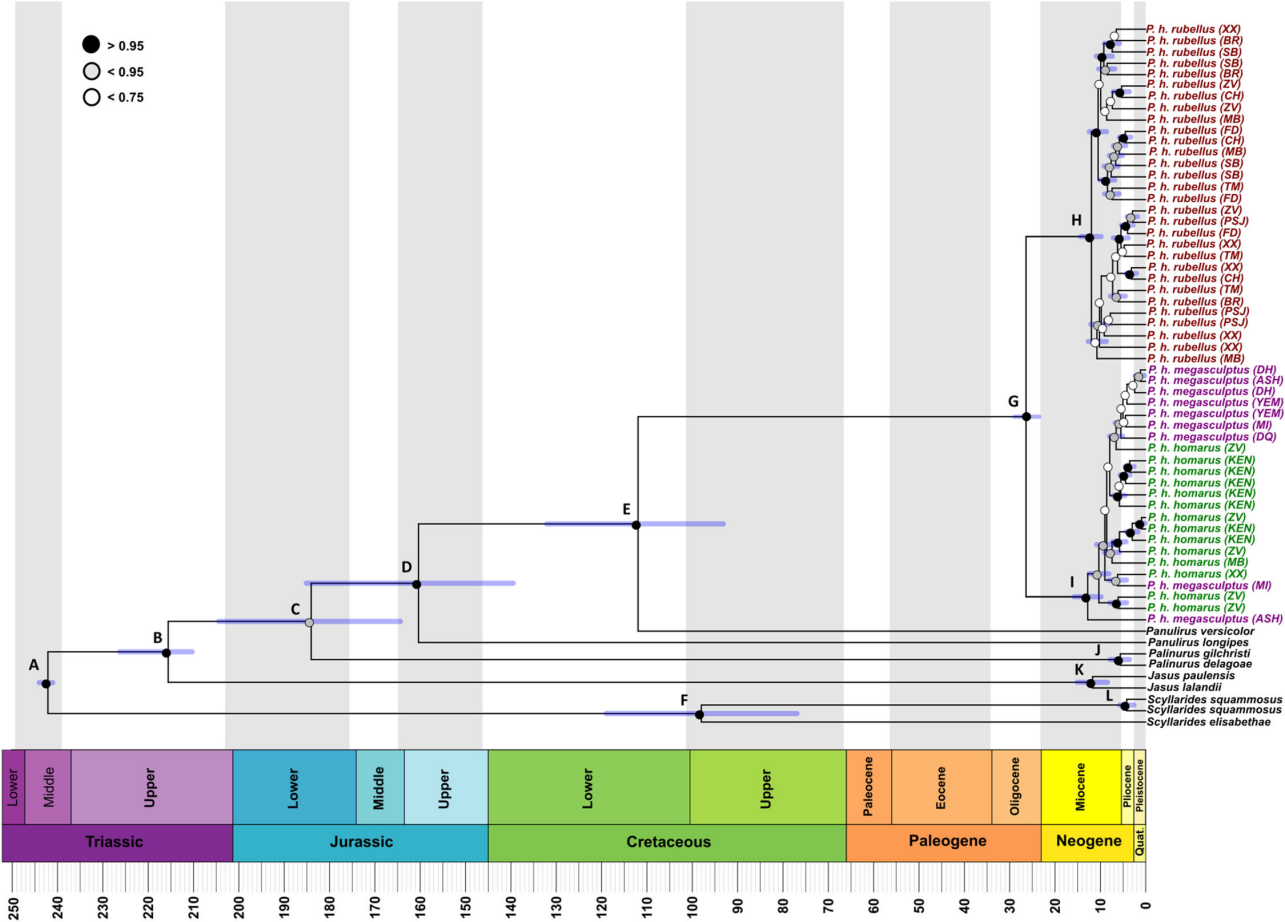
BEAST maximum clade credibility tree inferred from the supermatrix analysis with fossil calibrated nodes. Colored circles on the nodes indicate Bayesian posterior probability support. Letters on the nodes correspond to Table 3. Shaded bars indicate the 95% highest posterior density (HPD) credibility intervals which are listed in Table 3.

The maximum clade probability tree generated using BEAST with divergence times based on fossil calibration points is congruent with the four-gene concatenated phylogeny inferred using ML and BI methods. *Panulirus homarus homarus* and *Panulirus homarus rubellus* were recovered as two distinct monophyletic groups with high posterior probability of 1.0 (Fig. 3). *Panulirus homarus megasculptus* clustered with *Panulirus homarus homarus* individuals. *Panulirus homarus rubellus* and *Panulirus homarus homarus* last shared a common ancestor during the Oligocene, approximately 26 MYA (95% HPD 23.6–29.5, Table 3). The divergence times of the other species used as outgroups are consistent with those found by other studies (Bracken-Grissom et al., 2014; Palero et al., 2009; Tourinho, Solé-Cava & Lazoski, 2012).

**Table 3:** Lobster divergence dates estimated using fossil calibrated nodes.

Label	Node	Mean	95% HPD (MYA)
A	Achelata	242	241.0–244.0
B	Palinuridae	215	210.3–226.3
C	*Palinurus*	184	164.3–204.7
D	*Panulirus*	160	139.5–185.1
E	*Panulirus*	111	93.2–132.1
F	*Scyllarides elisabethae*	98	76.9–119
G	*Panulirus homarus homarus subspecies complex*	26	23.6–29.5
H	*Panulirus homarus rubellus*	12.7	9.8–15.7
I	*Panulirus homarus homarus*	11.9	9.8–14.9
J	*Palinurus delagoae & Palinurus gilchristi*	5.6	3.5–7.8
K	*Jasus lalandii & Jasus paulensis*	11.7	8.5–15.1
L	*Scyllarides squammosus*	4.2	2.6–5.7

**Figure 4:**
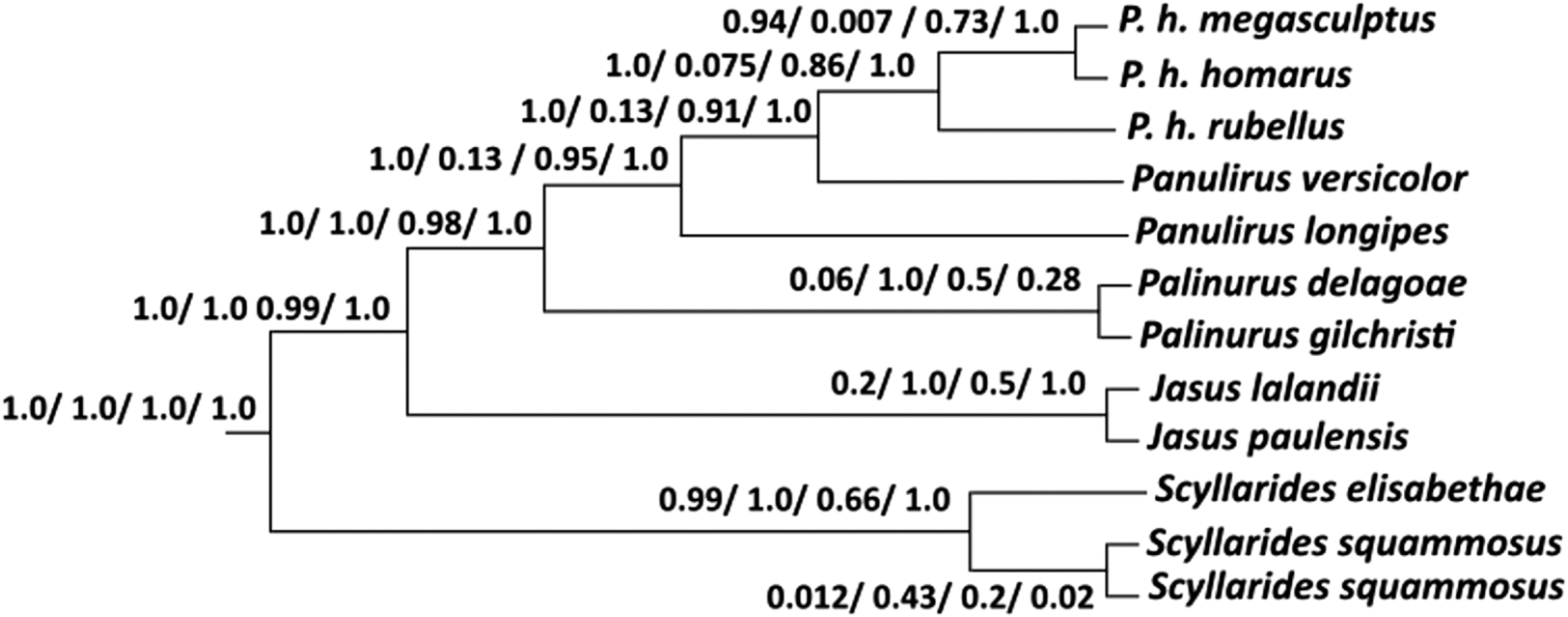
BPP Tree. BP & P majority rule consensus tree obtained using the BEAST guide tree and rjMCMC algorithm one (species delimitation using a fixed guide tree) showing Bayesian posterior probability values for the delimitation of species for each of the different prior combinations.

The choice of prior distributions seemed to influence species delimitation. Prior combinations 1 and 4 resulted in high posterior probability support for *Panulirus homarus rubellus* as a separate species (BPP = 1.0, Fig. 4). Prior combination 3 yielded moderate support (BPP = 0.86, Fig. 4) while the prior combination specifying a deep divergence and small ancestral population size resulted in no support for *Panulirus homarus rubellus* as a separate species. The prior combination 4 supported the distinction of *Panulirus homarus homarus* and *Panulirus homarus megasculptus* (BPP = 1.0, Fig. 4). BP & P consistently delimited *Panulirus longipes and Panulirus versicolor* as distinct species (BPP >0.90, Fig. 4), except for prior combination 2 (BPP = 0.13) and confirmed that the two *Scyllarides squammosus* individuals were not separate species (BPP <0.50, Fig. 4). Only prior combination 3 did not support *Scyllarides elisabethae* as being a distinct species (BPP = 0.66, Fig. 4). Interestingly, there was low support for the separation of *Jasus paulensis* and *Jasus lalandii* (BPP = 0.2 & 0.5, Fig. 4) under prior combinations 1 and 3. There was also low support for the separation of *Palinurus delagoae* and *Palinurus gilchristi* (BPP = 0.06, 0.5 & 0.28, Fig. 4) under prior combinations 1, 3 and 4.

## Discussion

This study incorporated evidence from molecular (mtDNA and ncDNA), morphology and fossil information to explore the phylogeny of the *Panulirus homarus* subspecies complex throughout the Indo-West Pacific. An important question addressed was whether the *Panulirus homarus rubellus* subspecies, occurring along the Southeast African coast and Madagascar, was an independently evolving lineage. The results from this study, using more individuals from a wider geographic range, and additional analyses, corroborate the findings of Lavery et al. (2014). They recognized *Panulirus homarus rubellus* as being a distinct lineage. In addition, genetic differences found in this study between *Panulirus homarus homarus* and the Arabian Sea *Panulirus homarus megasculptus* was not substantial enough to warrant the subspecies classification and we suggest that these taxa represent a single morphologically polymorphic lineage. We suggest that *Panulirus homarus rubellus* be elevated to species level, and named *Panulirus rubellus*, or the African spiny lobster, under the universal species concept. According to this concept, the only defining property of the species is being a separately evolving metapopulation lineage, and the other species concepts are treated as secondary defining characteristics (de Queiroz, 2007). The *Panulirus homarus rubellus* lineage fulfils the secondary defining criteria for the morphological (Mishler, 1985; Nelson & Plantick, 1981), genotypic (Mallet, 1995) and phylogenetic (Cracraft, 1983) species concepts.

At the individual gene level, nuclear gene trees did not support the separation of the subspecies as the trees were unresolved (Figs. S3 and S4). When all four genes were analyzed in combination, phylogenetic signal increased along with ML bootstrap and BPP support (Fig. 2). Incongruence between phylogenies produced using only single gene datasets is a challenge in molecular phylogenetics. To circumvent this issue, many studies now use combined genetic data instead of relying on single gene trees to represent the species tree. Studies have demonstrated that analyses using several genes concatenated can reveal character support for relationships in the overall tree from data sets which on their own do not support the relationships. Concatenated trees can increase discriminatory power and phylogenetic signal (Olmstead & Sweere, 1994; Gatesy, O’ Grady & Baker, 1999; Willows-Munro, Matthee & Robinson, 2005; de Queiroz & Gatesy, 2007).

Between the three *Panulirus homarus* subspecies, the uncorrected pairwise distance for COI was 4.8% in our study, compared to the 9% estimate of Lavery et al. (2014). Our estimate was, however, based on a much larger sample size (44 sequences) than in the previous study (seven sequences), which could explain the difference. The pairwise distance for CR in our study of 25.8% was comparable to the 30% of Lavery et al. (2014), thus providing further confidence in the results. For the nuclear markers, pairwise distances between the subspecies was as expected, higher than what was obtained for the COI marker, 8.3% for β-tubulin and 7.7% for ITS-1.

The question then arises of how *Panulirus homarus rubellus* (in the south) and *Panulirus homarus homarus* (further north) diverged, from both life history and oceanographic perspectives. *Panulirus homarus* has a long planktonic larval phase which may drift in ocean currents for many months (Berry, 1974a), during which they can be dispersed over long distances. Phyllosoma larvae can also swim actively to position themselves in the water column, or facilitate dispersal or return them to coastal settlement areas (Phillips et al., 2006). In spite of high dispersal potential of larvae, potentially facilitating larval mixing and genetic connectivity (Siegel et al., 2003), dispersal patterns can be constrained by larval retention in semi-permanent gyres or current systems, which, in turn, are affected by climate change (Pollock, 1993; Cowen & Sponaugle, 2009).

Based on the signal from our genetic analysis, we speculate that *Panulirus homarus rubellus* larvae are constrained to the southern part of the Southwest Indian Ocean when they become trapped within inshore ocean gyres of the Mozambique Channel and over the Southeast African shelf (Reddy et al., 2014). Larvae that stray further offshore, and become entrained in the Agulhas Current will be swept southwestwards and lost. A similar scenario was proposed for another spiny lobster species (*Palinurus gilchristi*) in the same region, in which larvae retained over the shelf and the Agulhas Bank, between the Agulhas Current and the coast, would remain viable, whereas those caught up in the Current would be lost (Groeneveld & Branch, 2002; Tolley et al., 2005). In Southern Australia, larvae of a coastal broadcast spawner that remain on the continental shelf (where currents are erratic and often shoreward), returned to the coast in much larger numbers than those entrained in shelf-edge boundary currents (Teske et al., 2015). We propose that similar source and sink mechanisms act to constrain *Panulirus homarus rubellus* to coastal areas in the Southwest Indian Ocean. Adult *Panulirus homarus homarus* occur sympatrically along the Southeast African coast, at a low rate, possibly because of larval spill-over from further north in the Mozambique Channel, facilitated by surface drift resulting from monsoon winds (Pollock, 1993).

New species may arise if larval retention mechanisms persist, separating species geographically, and in time leading to reproductive isolation (Pollock, 1995). In the present study, there was no evidence of hybridization between *Panulirus homarus homarus* and *Panulirus homarus rubellus*. The occurrence of hybrids between *Panulirus homarus homarus* and *Panulirus homarus rubellus* was first reported by Berry (1974b), in a boundary area where both subspecies occurred (Southern Mozambique), and where the frequency of *Panulirus homarus homarus* increases and that of *Panulirus homarus rubellus* tapers off. This region could be a contact zone where *Panulirus homarus homarus* and *Panulirus homarus rubellus* individuals may interbreed, after secondary contact. The single case of hybridization found by Lavery et al. (2014) also highlights that mating can occur between them, but given the highly significant genetic differentiation between the two subspecies, it occurs at a low rate. van der Meeren, Chandrapavan & Breithaupt (2008) also showed that while mating is possible between clawed lobsters *Homarus americanus* and *Homarus gammarus*, the preference is toward conspecifics.

Allopatric speciation, or complete isolation between *Panulirus homarus homarus* and *Panulirus homarus rubellus* may not occur due to the dynamic nature of the ocean and few barriers to dispersal (Lessios, Kessing & Robertson, 1998; Waples, 1998). Rather, parapatric speciation (partial isolation) may be responsible for the genetic distinctiveness between them (Rocha & Bowen, 2008). The patterns in the genetic data correspond with the model of parapatric speciation (Wu & Ting, 2004). For example, mitochondrial COI is under strong selection and thus segregates first, whilst the other markers that are not under such selective pressure move between incipient species until complete separation is achieved (Wu & Ting, 2004). The factors that promote parapatric speciation are characteristic of the *Panulirus homarus* subspecies complex, as they have a relatively wide geographic range, temporal and spatial differences in their ecological conditions and there is a reduction of effective migration rates between the subspecies because of local ocean currents, eddies and gyres (Coyne & Orr, 2004). The influence of oceanographic features and ecological factors such as sea temperature, salinity and turbidity on the distribution differences, speciation and genetic diversity between *Panulirus homarus homarus* and *Panulirus homarus rubellus* warrant further investigation.

The timing of the emergence of *Panulirus* in the early Mesozoic with divergence dating tree using the multilocus dataset and fossil calibrations is consistent with morphological evidence within the genus (George & Main, 1967). Using a divergence rate of 1% for COI suggests an estimated divergence of nine MYA for *Panulirus homarus rubellus* (Lavery et al., 2014). Estimates in this study using fossil calibrated nodes and four loci demonstrate that *Panulirus homarus rubellus* might have arisen between 10–16 MYA. George (2006) suggests that the fragmentation of the Tethys Sea is responsible for the radiation of *Panulirus*. Studies have shown that the final closure of the Tethys seaway, 14 MYA, during the Middle Miocene, had a significant impact on global ocean circulation (Hamon et al., 2013). Other investigations using marine isotopic data indicated that heat was transported from the Northern Indian Ocean to the Southern Ocean by a warm, saline water mass known as the Tethyan Indian Saline Water mass, and then ended due to the Tethys Sea closure (Flower & Kennett, 1994; Ramsay, Smart & Zachos, 1998; Woodruff & Savin, 1989). Modelling studies show that during this time, the closure involved changes in salinity and temperature in the Indian Ocean, leading to changes in latitudinal density gradient (Hamon et al., 2013). These oceanographic factors could have had an impact on the formation and speciation of *Panulirus homarus* given that they arose around this period.

The BP & P posterior probability results were dependent on choosing appropriate prior combinations, as observed in other studies (McKay et al., 2013; Zhang, Zhang & Yang, 2011). We propose that the most biologically relevant prior combination would be a small ancestral population size and shallow divergence (prior combination 3) because recently evolved lobsters such as the *Panulirus homarus* subspecies and *Panulirus versicolor* have lower levels of sequence divergence and shorter branch lengths between species, than between more ancestral species such as *Panulirus longipes* (Ptacek et al., 2001). This result provides further evidence that the taxonomy of *Panulirus homarus rubellus* should be reviewed.

To conclude, we used classical multilocus phylogenetic, coalescent-based and divergence time estimation with fossil calibration methods to resolve the *Panulirus homarus* subspecies complex. The lack of haplotypes shared between *Panulirus homarus homarus* and *Panulirus homarus rubellus*, and their distinct groupings on the four-gene concatenated phylogeny suggest that they are genetically distinct lineages. The observed genetic differentiation could be attributed to local larval retention mechanisms and ocean currents affecting dispersal capability of their long-lived phyllosoma stage. Based on the morphological (Berry, 1971) and distribution (Holthuis, 1991) differences and the results from the present study using a concatenated dataset of four genes—which strongly support the findings of Lavery et al. (2014), the taxonomic status of *Panulirus homarus rubellus* as a subspecies of *Panulirus homarus* should be re-evaluated. We suggest that it is acknowledged as a separately evolving lineage and a new species, *Panulirus rubellus,* from the Southwest Indian Ocean.

## Supplemental Information

10.7717/peerj.3356/supp-1 Supplemental Information 1 Table S1. List of *P. homarus* subspecies and outgroup taxa used for phylogenetic analyses.

10.7717/peerj.3356/supp-2 Supplemental Information 2 Table S2. Uncorrected pairwise distances for COI with standard errors. Uncorrected pairwise distances for COI (below the diagonal) with standard error estimates (above the diagonal) between the *P. homarus* subspecies and outgroups.

10.7717/peerj.3356/supp-3 Supplemental Information 3 Table S3. Uncorrected pairwise distances for CR with standard errors. Uncorrected pairwise distances for CR (below the diagonal) and standard error estimates (above the diagonal) between the *P. homarus* subspecies and outgroups.

10.7717/peerj.3356/supp-4 Supplemental Information 4 Table S4. Uncorrected pairwise distances for β-tubulin with standard errors. Uncorrected pairwise distances for β-tubulin (below the diagonal) and standard error estimates (above the diagonal) between the *P. homarus* subspecies and outgroups.

10.7717/peerj.3356/supp-5 Supplemental Information 5 Table S5. Uncorrected pairwise distances for ITS-1 with standard error estimates. Uncorrected pairwise distances for ITS-1 (below the diagonal) and standard error estimates (above the diagonal) between the *P. homarus* subspecies and outgroups.

10.7717/peerj.3356/supp-6 Supplemental Information 6 Fig. S.1. COI. (A) Maximum likelihood tree inferred from COI sequence data using only ingroup taxa and (B) with outgroup taxa. Maximum likelihood bootstrap support values and Bayesian posterior probabilities are indicated on the nodes. The colors represent the different subspecies. (C) Median-joining haplotype network for COI, constructed using PopArt 1.7. * indicates no bootstrap support.

10.7717/peerj.3356/supp-7 Supplemental Information 7 Fig. S.2. CR. (A) Maximum likelihood tree inferred from CR sequence data using only ingroup taxa and (B) with the outgroup taxa. Maximum likelihood bootstrap support values and Bayesian posterior probabilities are indicated on the nodes. The colors represent the different subspecies. (C) Median-joining haplotype network for CR, constructed using PopArt 1.7. * indicates no bootstrap support.

10.7717/peerj.3356/supp-8 Supplemental Information 8 Fig. S.3. BT. (A) Maximum likelihood tree inferred from β-tubulin sequence data using only ingroup taxa and (B) with the outgroup taxa. Maximum likelihood bootstrap support values and Bayesian posterior probabilities are indicated on the nodes. The colors represent the different subspecies. * indicates no bootstrap support.

10.7717/peerj.3356/supp-9 Supplemental Information 9 Fig. S.4. ITS. (A) Maximum likelihood tree inferred from ITS-1 sequence data using only ingroup taxa and (B) with the outgroup taxa. Maximum likelihood bootstrap support values and Bayesian posterior probabilities are indicated on the nodes. The colors represent the different subspecies. * indicates no bootstrap support.

10.7717/peerj.3356/supp-10 Supplemental Information 10 Fig. S.5. Mitochondrial. (A) Maximum likelihood tree inferred from the combined mitochondrial (COI + CR) sequence data using only ingroup taxa and (B) with the outgroup taxa. Maximum likelihood bootstrap support values and Bayesian posterior probabilities are indicated on the nodes. The colors represent the different subspecies. * indicates no bootstrap support.

10.7717/peerj.3356/supp-11 Supplemental Information 11 Fig. S.6. Nuclear. (A) Maximum likelihood tree inferred from the combined nuclear (β-tubulin + ITS-1) sequence data using only ingroup taxa and (B) with the outgroup taxa. Maximum likelihood bootstrap support values and Bayesian posterior probabilities are indicated on the nodes. The colors represent the different subspecies. * indicates no bootstrap support.

10.7717/peerj.3356/supp-12 Supplemental Information 12 COI dataset.

10.7717/peerj.3356/supp-13 Supplemental Information 13 CR dataset.

10.7717/peerj.3356/supp-14 Supplemental Information 14 BTUB dataset.

10.7717/peerj.3356/supp-15 Supplemental Information 15 ITS dataset.

## Additional Information and Declarations

### Field Study Permissions

The following information was supplied relating to field study approvals (i.e., approving body and any reference numbers): The Department of Environmental Affairs and the Department of Agriculture, Forestry & Fisheries granted approval for this study. Samples from other countries were obtained from research institutes there, under their rules and regulations: Instituto Nacional de Investigação Pesqueira, Maputo, Moçambique. Ministry of Agriculture and Fisheries, Marine Science and Fisheries Centre, Oman.

### DNA Deposition

The following information was supplied regarding the deposition of DNA sequences: All sequences used in this study are listed with their GenBank accession numbers and sources in Table S1. We have included the sequence alignment files in the Supplemental Data.

### Data Availability

The following information was supplied regarding data availability: The raw data has been supplied as Supplemental Dataset Files.
