# Supplementary material for: A molecular phylogeny of the spiny lobster Panulirus homarus highlights a separately evolving lineage from the Southwest Indian Ocean
Source: PeerJ. 2017 May 25;5:e3356. doi: 10.7717/peerj.3356 (PMC5446773; doi:10.7717/peerj.3356)
Supplement: Supplemental Information 1 [file peerj-05-3356-s001.docx]

Table S1. List of *P. homarus* subspecies and outgroup taxa used for phylogenetic analyses.

| **Specimen ID** | **Identification** | **Location** | **COI** | **CR** | **BTUB** | **ITS-1** | **Reference** |
| --- | --- | --- | --- | --- | --- | --- | --- |
| **KB01** | *P. h. homarus* | Kenya | **KX275311** | **KX349875** | **KX397099** | **KX349818** | This study |
| **KB02** | *P. h. homarus* | Kenya | **KX275312** | **KX349876** | **KX397100** | **KX349819** | This study |
| **KB08** | *P. h. homarus* | Kenya | **KX275313** | **KX349877** | **KX397101** | **KX349820** | This study |
| **KB10** | *P. h. homarus* | Kenya | **KX275314** | **KX349878** | **KX397102** | **KX349821** | This study |
| **SB10** | *P. h. rubellus* | Scottburgh, South Africa | **KX275316** | **KX349826** | **KX397046** | **KX349757** | This study |
| **SB11** | *P. h. rubellus* | Scottburgh, South Africa | **KX275317** | **KX349827** | **KX397047** | **KX349758** | This study |
| **SB12** | *P. h. rubellus* | Scottburgh, South Africa | **KX275318** | **KX349828** | **KX397048** | **KX349759** | This study |
| SB14 | *P. h. rubellus* | Scottburgh, South Africa | KX275319 | * | KX397049 | KX349760 | This study |
| SB16 | *P. h. rubellus* | Scottburgh, South Africa | KX275320 | KX349829 | KX397050 | KX349761 | This study |
| **FD3** | *P. h. rubellus* | Fort Dauphin, Madagascar | **KX275321** | **KX349862** | **KX397085** | **KX349799** | This study |
| FD04 | *P. h. rubellus* | Fort Dauphin, Madagascar | KX275322 | * | * | KX349800 | This study |
| **FD7** | *P. h. rubellus* | Fort Dauphin, Madagascar | **KX275323** | **KX349859** | **KX397086** | **KX349801** | This study |
| **FD8** | *P. h. rubellus* | Fort Dauphin, Madagascar | **KX275324** | **KX349860** | **KX397087** | **KX349802** | This study |
| **BR1** | *P. h. rubellus* | Blood Reef, South Africa | **KX275326** | **KX349841** | **KX397074** | **KX349781** | This study |
| **BR3** | *P. h. rubellus* | Blood Reef, South Africa | **KX275327** | **KX349842** | **KX397075** | **KX349782** | This study |
| **BR4** | *P. h. rubellus* | Blood Reef, South Africa | **KX275328** | **KX349843** | **KX397076** | **KX349783** | This study |
| **CH5** | *P. h. rubellus* | Chidenguele, Mozambique | **KX275331** | **KX349825** | **KX397053** | **KX349775** | This study |
| CH7 | *P. h. rubellus* | Chidenguele, Mozambique | KX275332 | * | KX397054 | KX349776 | This study |
| **CH2** | *P. h. rubellus* | Chidenguele, Mozambique | **KX275333** | **KX349823** | **KX397051** | **KX349773** | This study |
| **CH3** | *P. h. rubellus* | Chidenguele, Mozambique | **KX275334** | **KX349824** | **KX397052** | **KX349774** | This study |
| MB2 | *P. h. rubellus* | Mdumbi, South Africa | KX275335 | KX349835 | KX397079 | KX349786 | This study |
| MB4 | *P. h. homarus* | Mdumbi, South Africa | KX275336 | KX349836 | KX397080 | KX349787 | This study |
| MB5 | *P. h. rubellus* | Mdumbi, South Africa | KX275337 | KX349837 | KX397081 | KX349788 | This study |
| **MB6** | *P. h. rubellus* | Mdumbi, South Africa | **KX275338** | **KX349838** | **KX397082** | **KX349789** | This study |
| **MB7** | *P. h. rubellus* | Mdumbi, South Africa | **KX275339** | **KX349839** | **KX397083** | **KX349790** | This study |
| **MB8** | *P. h. rubellus* | Mdumbi, South Africa | **KX275340** | **KX349840** | **KX397084** | **KX349791** | This study |
| PSJ1 | *P. h. rubellus* | Port St. Johns, South Africa | KX275341 | KX349830 | * | KX349777 | This study |
| PSJ2 | *P. h. rubellus* | Port St. Johns, South Africa | KX275342 | KX349831 | * | KX349778 | This study |
| PSJ5 | *P. h. rubellus* | Port St. Johns, South Africa | KX275343 | KX349832 | * | KX349779 | This study |
| PSJ7 | *P. h. rubellus* | Port St. Johns, South Africa | KX275344 | KX349833 | KX397073 | * | This study |
| PSJ9 | *P. h. rubellus* | Port St. Johns, South Africa | KX275345 | KX349834 | * | KX349780 | This study |
| TM01 | *P. h. rubellus* | Tinley Manor, South Africa | KX275347 | * | * | KX349763 | This study |
| **TM2** | *P. h. rubellus* | Tinley Manor, South Africa | **KX275348** | **KX349849** | **KX397061** | **KX349762** | This study |
| **TM3** | *P. h. rubellus* | Tinley Manor, South Africa | **KX275349** | **KX349850** | **KX397062** | **KX349764** | This study |
| TM07 | *P. h. rubellus* | Tinley Manor, South Africa | KX275350 | * | KX397063 | KX349765 | This study |
| **XX1** | *P. h. rubellus* | Xai Xai, Mozambique | **KX275351** | **KX349844** | **KX397055** | **KX349767** | This study |
| **XX4** | *P. h. rubellus* | Xai Xai, Mozambique | **KX275352** | **KX349845** | **KX397056** | **KX349768** | This study |
| XX5 | *P. h. rubellus* | Xai Xai, Mozambique | KX275353 | * | KX397057 | KX349769 | This study |
| **XX6** | *P. h. homarus* | Xai Xai, Mozambique | **KX275354** | **KX349846** | **KX397058** | **KX349770** | This study |
| **XX9** | *P. h. rubellus* | Xai Xai, Mozambique | **KX275355** | **KX349847** | **KX397059** | **KX349771** | This study |
| **XX10** | *P. h. rubellus* | Xai Xai, Mozambique | **KX275356** | **KX349848** | **KX397060** | **KX349772** | This study |
| ZV01 | *P. h. homarus* | Zavora, Mozambique | KX275357 | * | KX397065 | KX349792 | This study |
| ZV02 | *P. h. homarus* | Zavora, Mozambique | KX275358 | * | * | * | This study |
| **ZV03** | *P. h. homarus* | Zavora, Mozambique | **KX275359** | **KX349852** | **KX397066** | **KX349793** | This study |
| **ZV04** | *P. h. homarus* | Zavora, Mozambique | **KX275360** | **KX349853** | **KX397067** | **KX349794** | This study |
| **ZV05** | *P. h. homarus* | Zavora, Mozambique | **KX275361** | **KX349854** | **KX397068** | **KX349795** | This study |
| ZV07 | *P. h. rubellus* | Zavora, Mozambique | KX275362 | KX349855 | KX397069 | * | This study |
| **ZV10** | *P. h. homarus* | Zavora, Mozambique | **KX275363** | **KX349856** | **KX397070** | **KX349796** | This study |
| **ZV14** | *P. h. rubellus* | Zavora, Mozambique | **KX275364** | **KX349857** | **KX397071** | **KX349797** | This study |
| **ZV15** | *P. h. rubellus* | Zavora, Mozambique | **KX275365** | **KX349858** | **KX397072** | **KX349798** | This study |
| OM3 | *P. h. megasculptus* | Dhalkhut, Oman | KX275366 | * | * | KX349810 | This study |
| OM6 | *P. h. megasculptus* | Dhalkhut, Oman | KX275367 | * | * | KX349811 | This study |
| **Ash08** | *P. h. megasculptus* | Al Ashkharah, Oman | **KX275368** | **KX349864** | **KX397097** | **KX349814** | This study |
| **Ash04** | *P. h. megasculptus* | Al Ashkharah, Oman | **KX275369** | **KX349863** | ***** | **KX349815** | This study |
| DQ08 | *P. h. megasculptus* | Duqm, Oman | KX275370 | * | * | * | This study |
| **M01** | *P. h. megasculptus* | Mirbat, Oman | **KX275371** | **KX349865** | **KX397095** | ***** | This study |
| **Yem02** | *P. h. megasculptus* | Yemen | **KX275372** | **KX349867** | **KX397093** | **KX349816** | This study |
| **Yem10** | *P. h. megasculptus* | Yemen | **KX275373** | **KX349868** | **KX397094** | **KX349817** | This study |
| **DQ07** | *P. h. megasculptus* | Duqm, Oman | **KX275374** | ***** | **KX397098** | **KX349812** | This study |
| **M11** | *P. h. megasculptus* | Mirbat, Oman | **KX275375** | **KX349866** | **KX397096** | **KX349813** | This study |
| **Ken04** | *P. h. homarus* | Kenya | **KX275376** | **KX349869** | **KX397089** | **KX349804** | This study |
| Ken07 | *P. h. homarus* | Kenya | KX275377 | * | * | KX349805 | This study |
| Ken08 | *P. h. homarus* | Kenya | KX275378 | * | KX397090 | KX349806 | This study |
| Ken10 | *P. h. homarus* | Kenya | KX275379 | * | KX397092 | KX349808 | This study |
| Ken13 | *P. h. homarus* | Kenya | KX275380 | KX349871 | * | KX349809 | This study |
| **J_lal** | *Jasus lalandii* | uShaka Marine World, South Africa | **KX275382** | **KX349872** | **KX397037** | **KX349748** | This study |
| **J_paul** | *Jasus paulensis* | uShaka Marine World, South Africa | **KX275383** | ***** | **KX397038** | **KX349749** | This study |
| **P_gil** | *Palinurus gilchristi* | uShaka Marine World, South Africa | **KX275384** | **KX349873** | **KX397040** | **KX349751** | This study |
| **P_long** | *Panulirus longipes* | uShaka Marine World, South Africa | **KX275385** | **KX349874** | **KX397041** | **KX349752** | This study |
| **P_versi** | *Panulirus vesicolor* | uShaka Marine World, South Africa | **KX275386** | ***** | **KX397042** | **KX349753** | This study |
| **SE1** | *Scyllarides elisabethae* | uShaka Marine World, South Africa | **KX275387** | ***** | **KX397044** | **KX349755** | This study |
| **SS1** | *Scyllarides squammosus* | Durban, South Africa | **KX275388** | ***** | **KX397045** | **KX349756** | This study |
| **SS2** | *Scyllarides squammosus* | Durban, South Africa | **KX275389** | ***** | **KX397043** | **KX349754** | This study |
| P_dela | *Palinurus delagoae* | uShaka Marine World, South Africa | * | * | KX397039 | KX349750 | This study |
| Ash01 | *P. h. megasculptus* | Al Ashkharah, Oman | KY860538 | * | * | * | This study |
| Ash03 | *P. h. megasculptus* | Al Ashkharah, Oman | KY860539 | * | * | * | This study |
| DQ06 | *P. h. megasculptus* | Duqm, Oman | KY860540 | * | * | * | This study |
| M02 | *P. h. megasculptus* | Mirbat, Oman | KY860541 | * | * | * | This study |
| M04 | *P. h. megasculptus* | Mirbat, Oman | KY860542 | * | * | * | This study |
| OM01 | *P. h. megasculptus* | Dhalkhut, Oman | KY860543 | * | * | KY860557 | This study |
| OM03 | *P. h. megasculptus* | Dhalkhut, Oman | KY860544 | * | * | * | This study |
| YEM04 | *P. h. megasculptus* | Yemen | KY860545 | * | * | * | This study |
| YEM05 | *P. h. megasculptus* | Yemen | KY860546 | * | * | * | This study |
| FD10 | *P. h. rubellus* | Fort Dauphin, Madagascar | KY860547 | KY860552 | * | KY860558 | This study |
| BR02 | *P. h. rubellus* | Blood Reef, South Africa | KY860548 | KY860553 | KY860562 | KY860559 | This study |
| BR05 | *P. h. rubellus* | Blood Reef, South Africa | KY860549 | KY860554 | KY860560 | KY860563 | This study |
| TM04 | *P. h. rubellus* | Tinley Manor, South Africa | KY860550 | KY860555 | KY860564 | * | This study |
| TM06 | *P. h. rubellus* | Tinley Manor, South Africa | KY860551 | KY860556 | KY860565 | KY860561 | This study |

* Sequence not obtained for that marker.

Accessions in BOLD indicate the individuals used for divergence dating and BP & P analyses.
