# Supplementary material for: A molecular phylogeny of the spiny lobster Panulirus homarus highlights a separately evolving lineage from the Southwest Indian Ocean
Source: PeerJ. 2017 May 25;5:e3356. doi: 10.7717/peerj.3356 (PMC5446773; doi:10.7717/peerj.3356)
Supplement: Supplemental Information 2 — Uncorrected pairwise distances for COI (below the diagonal) with standard error estimates (above the diagonal) between the P. homarus subspecies and outgroups. [file peerj-05-3356-s002.docx]

Table S2. Uncorrected pairwise distances for COI (below the diagonal) with standard error estimates (above the diagonal) between the *P. homarus* subspecies and outgroups.

|  | **1** | **2** | **3** | **4** | **5** | **6** | **7** | **8** |
| --- | --- | --- | --- | --- | --- | --- | --- | --- |
| **1. *P. h. megasculptus*** |  | 0.002 | 0.009 | 0.021 | 0.020 | 0.019 | 0.019 | 0.015 |
| **2. *P. h. homarus*** | 0.016 |  | 0.009 | 0.021 | 0.020 | 0.019 | 0.019 | 0.016 |
| **3. *P. h. rubellus*** | 0.049 | 0.047 |  | 0.020 | 0.020 | 0.019 | 0.018 | 0.015 |
| **4. *J. lalandii*** | 0.190 | 0.191 | 0.180 |  | 0.011 | 0.020 | 0.021 | 0.020 |
| **5. *J. paulensis*** | 0.203 | 0.203 | 0.193 | 0.071 |  | 0.021 | 0.022 | 0.019 |
| **6. *P. gilchristi*** | 0.180 | 0.178 | 0.183 | 0.179 | 0.184 |  | 0.022 | 0.019 |
| **7. *P. longipes*** | 0.183 | 0.183 | 0.185 | 0.209 | 0.207 | 0.217 |  | 0.018 |
| **8. *P. versicolor*** | 0.132 | 0.133 | 0.136 | 0.191 | 0.191 | 0.189 | 0.184 |  |
