# Supplementary material for: A molecular phylogeny of the spiny lobster Panulirus homarus highlights a separately evolving lineage from the Southwest Indian Ocean
Source: PeerJ. 2017 May 25;5:e3356. doi: 10.7717/peerj.3356 (PMC5446773; doi:10.7717/peerj.3356)
Supplement: Supplemental Information 3 — Uncorrected pairwise distances for CR (below the diagonal) and standard error estimates (above the diagonal) between the P. homarus subspecies and outgroups. [file peerj-05-3356-s003.docx]

Table S3. Uncorrected pairwise distances for CR (below the diagonal) and standard error estimates (above the diagonal) between the *P. homarus* subspecies and outgroups.

|  | **1** | **2** | **3** | **4** | **5** | **6** |
| --- | --- | --- | --- | --- | --- | --- |
| **1. *P. h. megasculptus*** |  | 0.014 | 0.058 | 0.069 | 0.072 | 0.055 |
| **2. *P. h. homarus*** | 0.035 |  | 0.056 | 0.067 | 0.071 | 0.056 |
| **3. *P. h. rubellus*** | 0.258 | 0.258 |  | 0.067 | 0.061 | 0.054 |
| **4. *P. gilchristi*** | 0.480 | 0.488 | 0.545 |  | 0.061 | 0.067 |
| **5. *P. longipes*** | 0.462 | 0.455 | 0.442 | 0.614 |  | 0.059 |
| **6. *J. lalandii*** | 0.526 | 0.525 | 0.595 | 0.632 | 0.526 |  |
