# Supplementary material for: A molecular phylogeny of the spiny lobster Panulirus homarus highlights a separately evolving lineage from the Southwest Indian Ocean
Source: PeerJ. 2017 May 25;5:e3356. doi: 10.7717/peerj.3356 (PMC5446773; doi:10.7717/peerj.3356)
Supplement: Supplemental Information 4 — Uncorrected pairwise distances for β-tubulin (below the diagonal) and standard error estimates (above the diagonal) between the P. homarus subspecies and outgroups. [file peerj-05-3356-s004.docx]

Table S4. Uncorrected pairwise distances for β-tubulin (below the diagonal) and standard error estimates (above the diagonal) between the *P. homarus* subspecies and outgroups.

|  | **1** | **2** | **3** | **4** | **5** | **6** | **7** | **8** | **9** |
| --- | --- | --- | --- | --- | --- | --- | --- | --- | --- |
| **1. *P. h. megasculptus*** |  | 0.011 | 0.011 | 0.020 | 0.020 | 0.019 | 0.019 | 0.017 | 0.018 |
| **2. *P. h. homarus*** | 0.086 |  | 0.009 | 0.019 | 0.018 | 0.016 | 0.019 | 0.016 | 0.017 |
| **3. *P. h. rubellus*** | 0.083 | 0.081 |  | 0.019 | 0.018 | 0.018 | 0.019 | 0.016 | 0.018 |
| **4. *J. lalandii*** | 0.156 | 0.151 | 0.154 |  | 0.014 | 0.022 | 0.022 | 0.021 | 0.021 |
| **5. *J. paulensis*** | 0.143 | 0.144 | 0.143 | 0.065 |  | 0.022 | 0.023 | 0.021 | 0.022 |
| **6. *P. delagoae*** | 0.179 | 0.163 | 0.172 | 0.174 | 0.162 |  | 0.018 | 0.022 | 0.021 |
| **7. *P. gilchristi*** | 0.168 | 0.163 | 0.165 | 0.178 | 0.182 | 0.113 |  | 0.020 | 0.023 |
| **8. *P. versicolor*** | 0.131 | 0.112 | 0.120 | 0.158 | 0.154 | 0.162 | 0.134 |  | 0.020 |
| **9. *P. longipes*** | 0.128 | 0.132 | 0.135 | 0.166 | 0.150 | 0.194 | 0.182 | 0.130 |  |
