# Supplementary material for: A molecular phylogeny of the spiny lobster Panulirus homarus highlights a separately evolving lineage from the Southwest Indian Ocean
Source: PeerJ. 2017 May 25;5:e3356. doi: 10.7717/peerj.3356 (PMC5446773; doi:10.7717/peerj.3356)
Supplement: Supplemental Information 5 — Uncorrected pairwise distances for ITS-1 (below the diagonal) and standard error estimates (above the diagonal) between the P. homarus subspecies and outgroups. [file peerj-05-3356-s005.docx]

Table S5. Uncorrected pairwise distances for ITS-1 (below the diagonal) and standard error estimates (above the diagonal) between the *P. homarus* subspecies and outgroups.

|  | **1** | **2** | **3** | **4** | **5** | **6** | **7** | **8** | **9** |
| --- | --- | --- | --- | --- | --- | --- | --- | --- | --- |
| **1. *P. h. megasculptus*** |  | 0.008 | 0.007 | 0.029 | 0.029 | 0.026 | 0.026 | 0.026 | 0.026 |
| **2. *P. h. homarus*** | 0.075 |  | 0.008 | 0.029 | 0.029 | 0.027 | 0.027 | 0.027 | 0.026 |
| **3. *P. h. rubellus*** | 0.077 | 0.068 |  | 0.029 | 0.029 | 0.026 | 0.026 | 0.026 | 0.026 |
| **4. *J. paulensis*** | 0.346 | 0.341 | 0.338 |  | 0.009 | 0.028 | 0.027 | 0.029 | 0.030 |
| **5. *J. lalandii*** | 0.345 | 0.337 | 0.337 | 0.028 |  | 0.028 | 0.028 | 0.030 | 0.029 |
| **6. *P. gilchristi*** | 0.296 | 0.287 | 0.291 | 0.280 | 0.298 |  | 0.003 | 0.027 | 0.030 |
| **7. *P. delagoae*** | 0.296 | 0.287 | 0.291 | 0.276 | 0.295 | 0.003 |  | 0.027 | 0.030 |
| **8. *P. longipes*** | 0.298 | 0.294 | 0.297 | 0.345 | 0.345 | 0.289 | 0.289 |  | 0.026 |
| **9. *P. versicolor*** | 0.361 | 0.354 | 0.353 | 0.435 | 0.438 | 0.429 | 0.429 | 0.382 |  |
