# Supplementary figures and images for: A molecular phylogeny of the spiny lobster Panulirus homarus highlights a separately evolving lineage from the Southwest Indian Ocean
Source: PeerJ. 2017 May 25;5:e3356. doi: 10.7717/peerj.3356 (PMC5446773; doi:10.7717/peerj.3356)

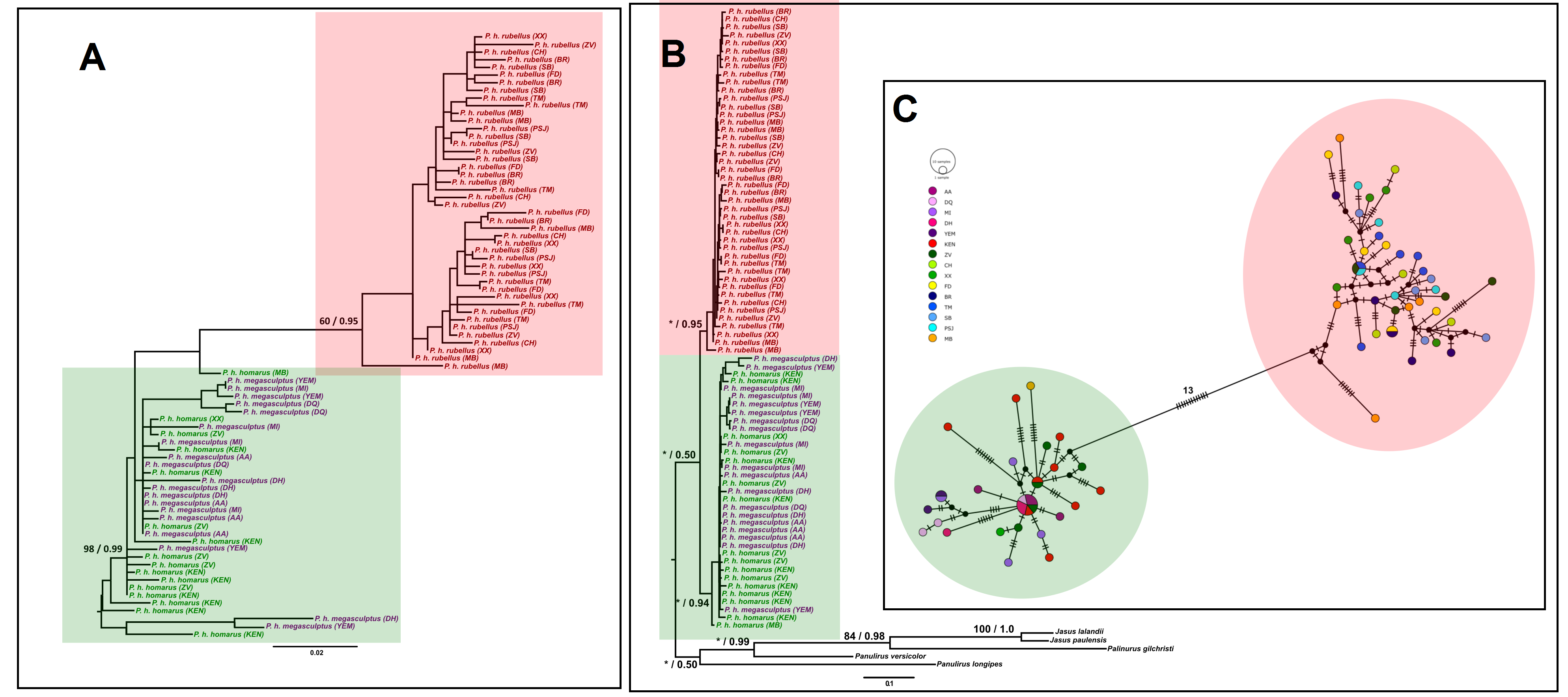

Supplement: Supplemental Information 6 — (A) Maximum likelihood tree inferred from COI sequence data using only ingroup taxa and (B) with outgroup taxa. Maximum likelihood bootstrap support values and Bayesian posterior probabilities are indicated on the nodes. The colors represent the different subspecies. (C) Median-joining haplotype network for COI, constructed using PopArt 1.7. * indicates no bootstrap support. [file peerj-05-3356-s006.png]

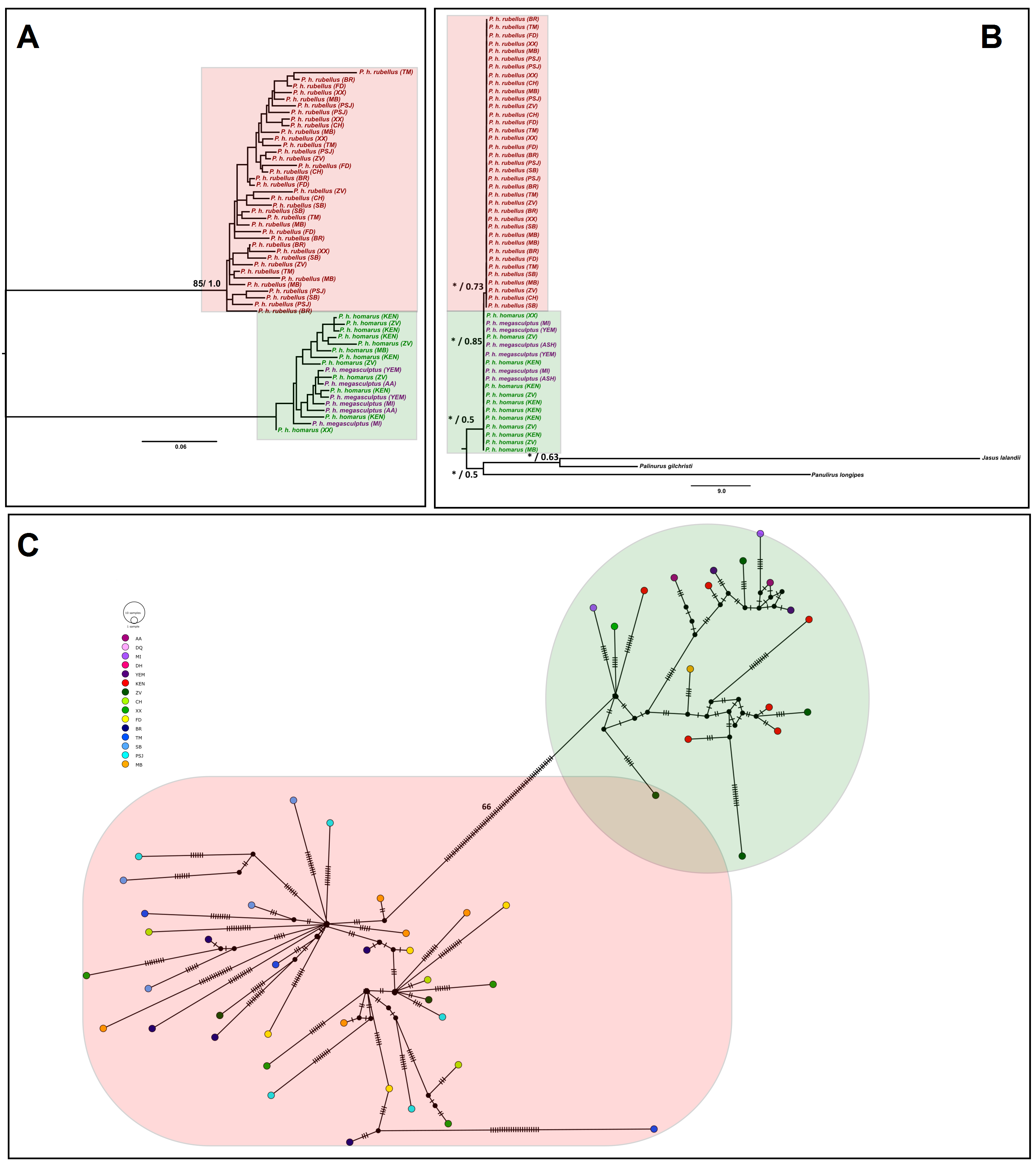

Supplement: Supplemental Information 7 — (A) Maximum likelihood tree inferred from CR sequence data using only ingroup taxa and (B) with the outgroup taxa. Maximum likelihood bootstrap support values and Bayesian posterior probabilities are indicated on the nodes. The colors represent the different subspecies. (C) Median-joining haplotype network for CR, constructed using PopArt 1.7. * indicates no bootstrap support. [file peerj-05-3356-s007.png]

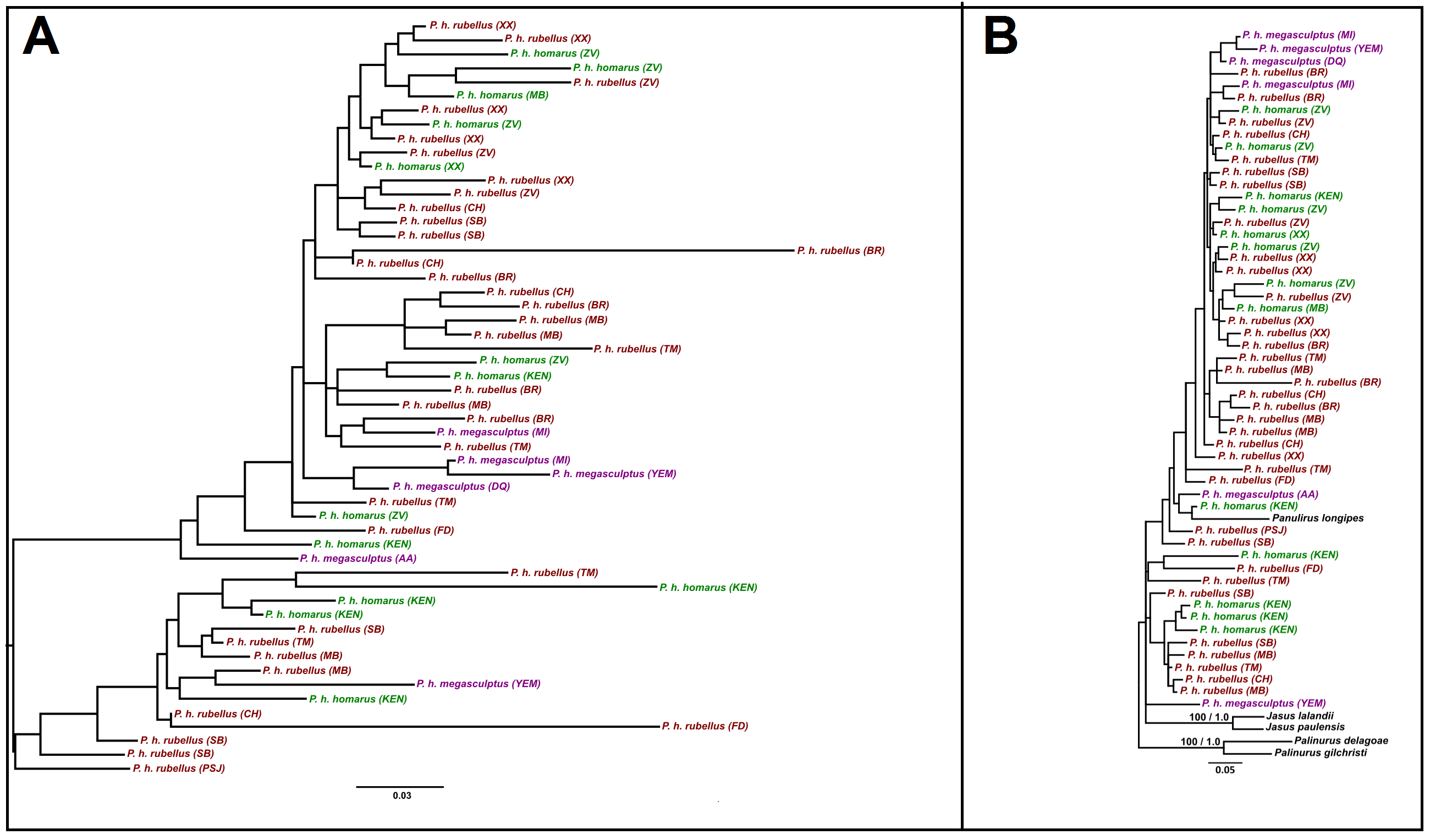

Supplement: Supplemental Information 8 — (A) Maximum likelihood tree inferred from β-tubulin sequence data using only ingroup taxa and (B) with the outgroup taxa. Maximum likelihood bootstrap support values and Bayesian posterior probabilities are indicated on the nodes. The colors represent the different subspecies. * indicates no bootstrap support. [file peerj-05-3356-s008.png]

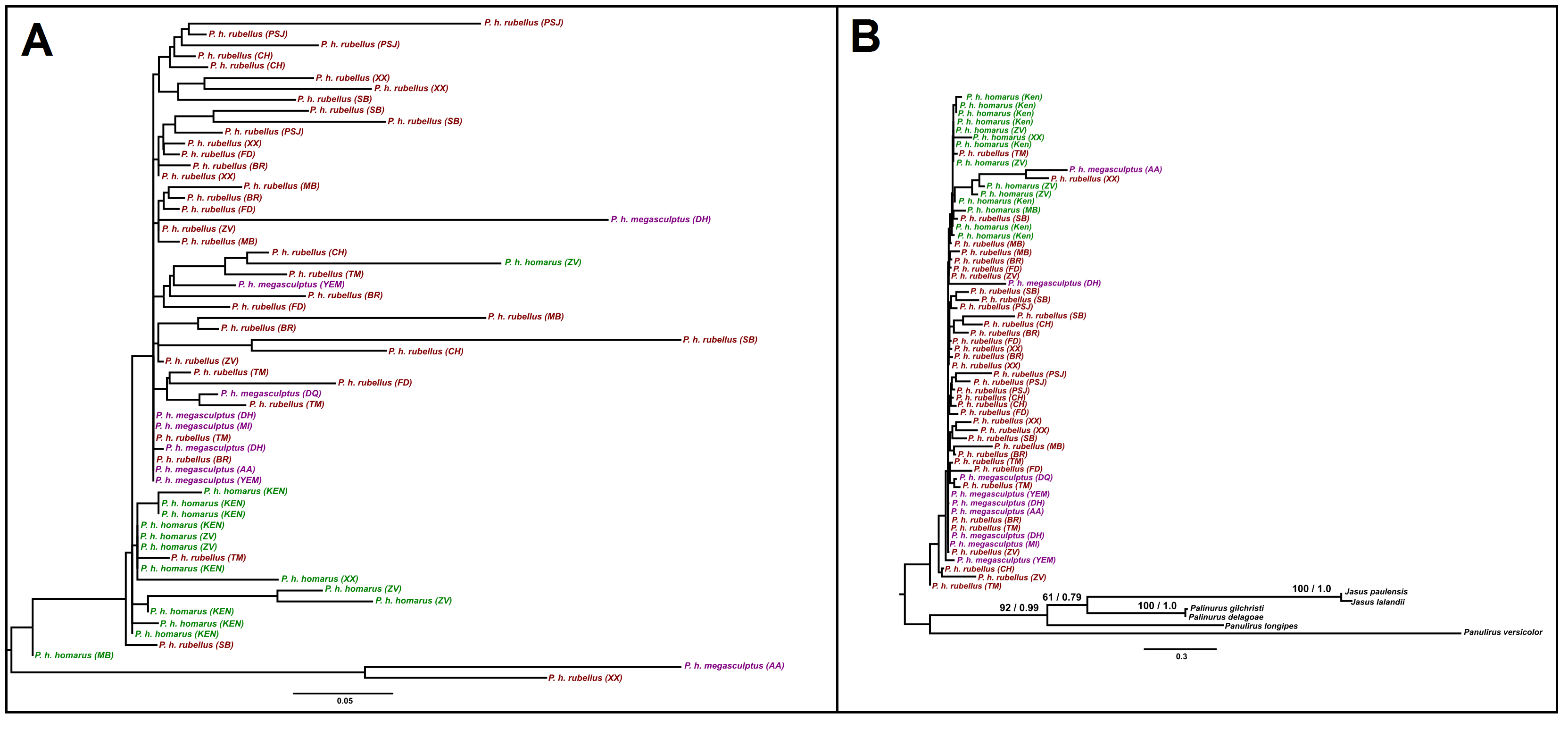

Supplement: Supplemental Information 9 — (A) Maximum likelihood tree inferred from ITS-1 sequence data using only ingroup taxa and (B) with the outgroup taxa. Maximum likelihood bootstrap support values and Bayesian posterior probabilities are indicated on the nodes. The colors represent the different subspecies. * indicates no bootstrap support. [file peerj-05-3356-s009.png]

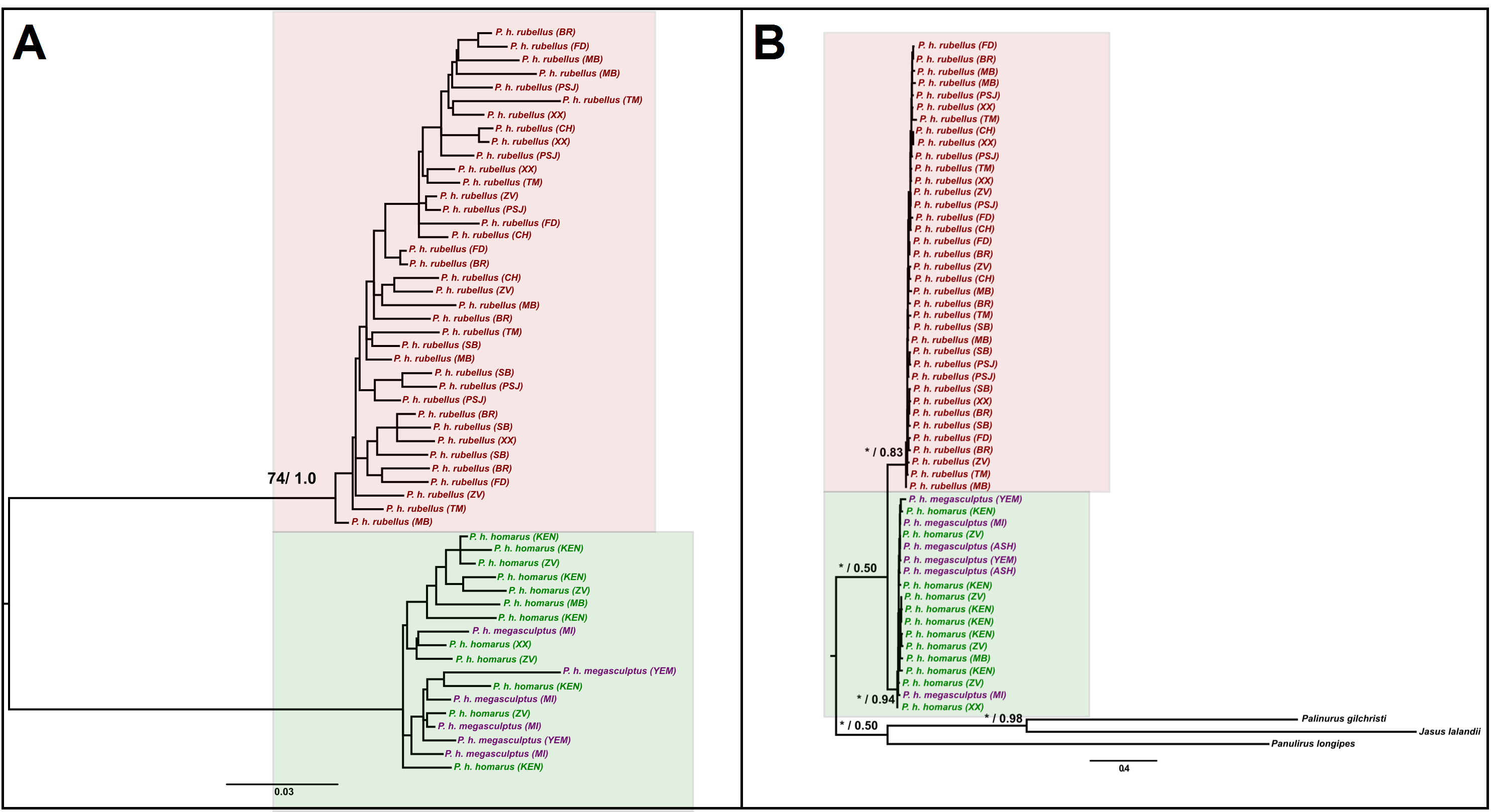

Supplement: Supplemental Information 10 — (A) Maximum likelihood tree inferred from the combined mitochondrial (COI + CR) sequence data using only ingroup taxa and (B) with the outgroup taxa. Maximum likelihood bootstrap support values and Bayesian posterior probabilities are indicated on the nodes. The colors represent the different subspecies. * indicates no bootstrap support. [file peerj-05-3356-s010.png]

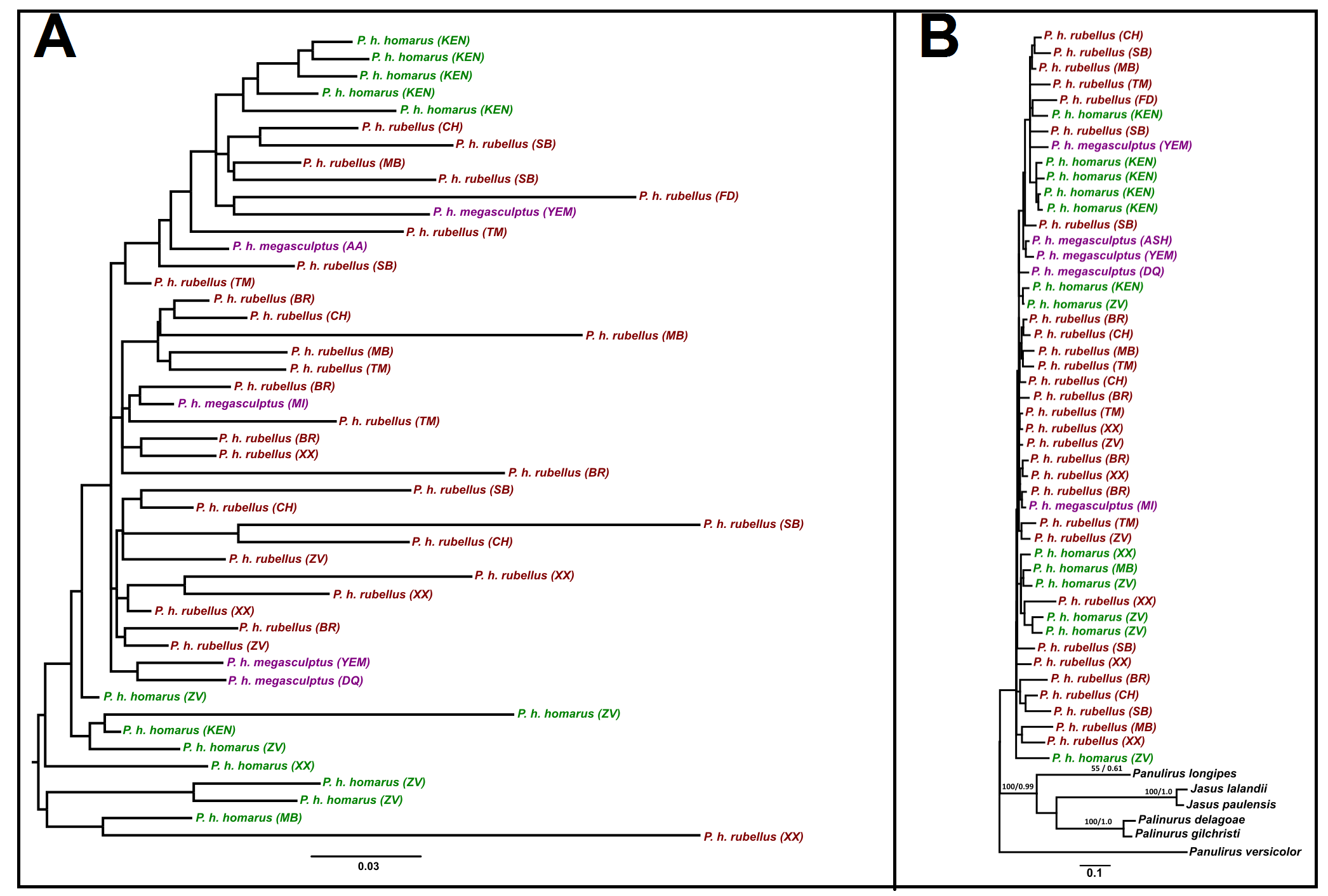

Supplement: Supplemental Information 11 — (A) Maximum likelihood tree inferred from the combined nuclear (β-tubulin + ITS-1) sequence data using only ingroup taxa and (B) with the outgroup taxa. Maximum likelihood bootstrap support values and Bayesian posterior probabilities are indicated on the nodes. The colors represent the different subspecies. * indicates no bootstrap support. [file peerj-05-3356-s011.png]
